# Optical linear systems framework for event sensing and computational neuromorphic imaging

**DOI:** 10.3389/fnins.2026.1831543

**Published:** 2026-05-19

**Authors:** Nimrod Kruger, Nicholas Owen Ralph, Gregory Cohen, Paul Hurley

**Affiliations:** 1 International Centre for Neuromorphic Systems, The MARCS Institute, Western Sydney University, Sydney, NSW, Australia; 2 Centre for Research in Mathematics and Data Science, Western Sydney University, Sydney, NSW, Australia

**Keywords:** computational imaging, dynamic linear system, event-vision sensors, neuromorphic engineering, Point Spread Function (PSF)

## Abstract

Event Vision Sensors, or neuromorphic cameras, report sparse, and asynchronous image change-related data and enable microsecond-scale sensing and high dynamic range, but challenge physics-based sensor design approaches. In response to log-intensity threshold-crossing instances, this event representation does not readily integrate with forward operators used to describe most computational imaging and optical systems. We present a physics-grounded processing pipeline that maps event streams to estimates of per-pixel log-intensity and its derivatives, and embeds these measurements in a dynamic linear systems model with a time-varying Point-Spread Function (PSF). This enables inverse filtering directly from event data via frequency-domain Wiener deconvolution with a known dynamic transfer function, enabling scene inference even when no image is formed on the sensor plane. We validated the approach in simulation for single and overlapping point-sources under modulated defocus, and on real event data from a tunable-focus telescope imaging a star field, demonstrating superior source localization and separability compared with frame-based imaging. The proposed framework provides a practical bridge between event sensing and model-based computational imaging for dynamic optical systems.

## 1 Introduction

An Event Vision Sensors (EVS) approximately represents asynchronous log-intensity changes $\frac{dL}{dt}$, where *L* ≡ *log*(*I*), rather than direct absolute intensity *I*, as is common for conventional frame-based sensors. In these complementary metal–oxide–semiconductor (CMOS) sensors, a timely per-pixel event of log-intensity threshold crossing is reported relative to an internal reference level, followed by updating that level in anticipation of the next crossing event. The ability of EVS to report changes at microsecond resolution and to achieve a high dynamic range compared to most image sensors is driving many event-based applications in computer vision. However, representing these events in a physics-grounded manner, so that their information can be processed using a dynamic linear systems approach, has not been thoroughly explored in the literature. A linear system description can be used to relate a full scene to the spatial-temporal event-stream output, informed by physical modeling of the optics and sensor, as is often used to design fast inference of a scene in frame-based computer-vision tasks. Currently, most EVS physics-grounded approaches first create a full image-from-events reconstruction, use the scene intensity constancy assumption, or both (Zhang et al., 2022). These typically assume the conventional pinhole camera model, with a sensor array that roughly maps the angular scene intensity, and are useful for recording through traditional in-focus imaging lenses. However, the pinhole camera model and scene intensity constancy assumption are not always applicable for a generalized imaging system.

The desire to recover more scene information in highly dynamic scenes, in applications such as robotics, industrial inspection, and medicine, has led to the proliferation of work using EVS for computational imaging. These include EVS plenoptic cameras (Qu et al., 2024), microscopy (Bian et al., 2024; Zhang et al., 2025; Baird et al., 2025), wavefront sensing (Kong et al., 2020), coded aperture (Shah et al., 2024; Habuchi et al., 2024), lens-less imaging (You et al., 2025), and more (Arja et al., 2025; Cai et al., 2025; Zhu et al., 2024a)—collectively coined Computational Neuromorphic Imaging (CNI) (Zhu et al., 2024b). This is an emerging field that aims to expand EVS to overcome the latency-bandwidth-sensitivity bottlenecks of traditional imaging, while maintaining low system costs, energy consumption, and data bandwidth.

Computational imaging system designs often use a forward operator that maps a transfer function from scene intensity, light field, or complex wavefront to the sensor readout signal. Scalar-theory methods (e.g., angular spectrum and Fresnel approximation) can be used to predict sensor response to scene illumination, and also design systems for extracting specific information under various assumptions and priors (Joseph, 1968). With event-stream's unique data structure of binary threshold crossing response to *log*-intensity and asynchronous sampling mode, the transfer function definition and the information we put through the system are not directly obtained. Consequently, current CNI designs often reuse optical methods proven for frame-based imaging, thereby reducing performance. Alternatively, successful data-driven approaches will be hard-pressed to extrapolate, missing model-based parameterization.

We propose a refreshed view of log-intensity change events in the context of linear optical systems, paving the way for CNI models and methods. This will expand the range of design possibilities for machine vision systems, microscopy, and advanced optical sensors. The following components are presented:

1. *Log-intensity derivative revisited:* we describe the relation between a pixel's log-intensity threshold-crossing event stream to the per-event estimate of log-intensity derivative, intensity, and intensity derivative, for the limited case of a simple event pixel model.
2. *Dynamic imaging system model:* describe a linear forward model for optical signals transferred through a system with internally controlled dynamics, represented by the operator *T*(*t*), and projected onto a sensor plane. The expected intensity and its changes are related to the information encoded in the event stream.
3. *Scene-informed spatial-temporal filter:* an example spatial-temporal transfer function is used to design a filter as an inverse operator on event-data to extract scene estimation.

A direct path from event-stream to scene inference is then facilitated by the combined use of event interpolation, known optical transfer functions (with controlled system parameters), and scene priors.

We demonstrated this path using a simulated optical model of an imaging system with a modulated focal length, and on real-world EVS data of a star field taken with a tunable liquid-lens imaging system. In these, the introduced controlled dynamics are achieved by the active focus and defocus of the Point-Spread Function of a static point source (or a star field) on the sensor plane. Presenting this framework with a dynamic system and a static scene allows us to use a simple operator for source estimation, implemented here as Wiener deconvolution on event-stream data. Previous research using active neuromorphic vision with a tunable liquid lens for point-source localization (Ralph et al., 2024) shows improved sensitivity, noise rejection, and localization error, compared to other EVS-based space imaging methods, but requires long accumulation time. The ability to perform a similar task via direct deconvolution from events presents an avenue for novel algorithms for this task and for various other CNI applications.

## 2 Methods

### 2.1 Notation and pixel level derivative estimation

**Figure 1:**
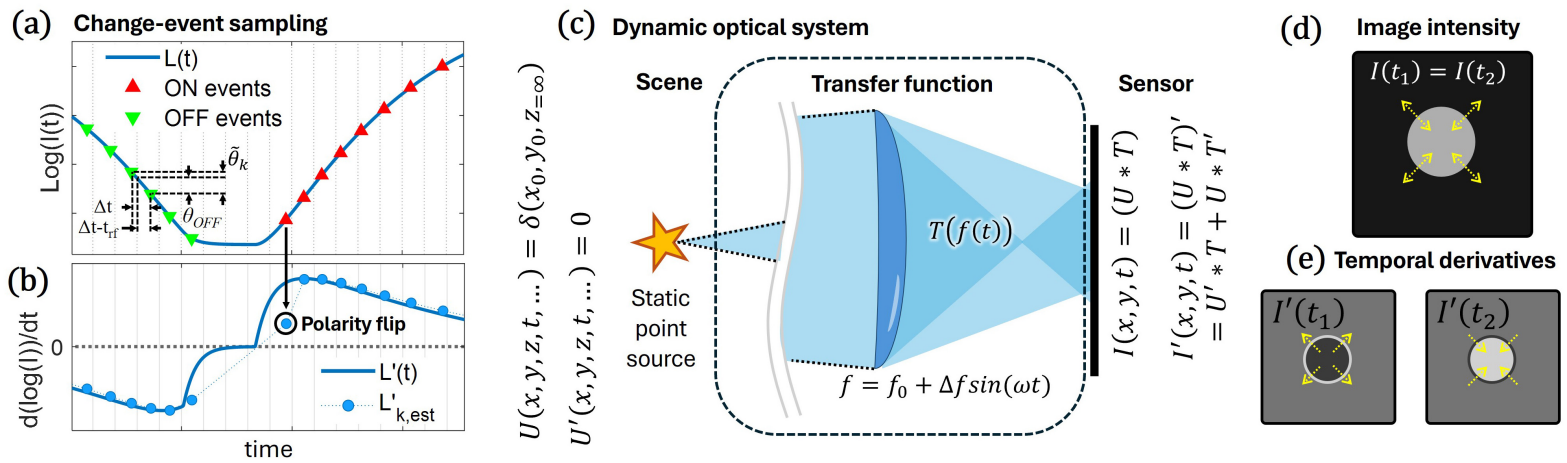
Threshold crossing events for dynamic optical systems. **(a)** The event-pixel typically reports the signal-threshold crossing of log-intensity, with a fixed refractory-period delay. **(b)** These are used to estimate the signal log-intensity derivative per event. A simple linear interpolation estimate works well for consecutive, similarly polarized events but fails for polarity-switching events. **(c)** A dynamic linear systems description of an optical system, *T*, projecting an input field *U* to an intensity map *I*. The product rule describes the temporal derivative of these functions. Such a system can dynamically model a point-source projection through an electrically tunable thin lens. **(d)** A depiction of the intensity map will show a spot changing size **(e)**, whereas its derivative for expansion at *t*_1_ or contraction at *t*_2_ ideally holds a uniform inter-spot value with an outer ring of opposite sign.

EVS's react to change in log-brightness by reporting either ON or OFF events when the pixel reference voltage crosses predefined θ_*ON*_ or θ_*OFF*_ threshold values, respectively, and then reset the pixel reference voltage after a pre-defined refractory period, *t*_*rf*_. The pixel voltage generally follows a logarithmic function of the impinging photon flux intensity. An event *e*_*k*_ ≡ (x_*k*_, *t*_*k*_, *p*_*k*_) represents such a change at pixel x_*k*_ = (*x*_*k*_, *y*_*k*_) at an instance *t*_*k*_, where *p*_*k*_ = 1 or −1 for log-intensity increase by θ_*ON*_ or decrease by θ_*OFF*_ respectively, assuming no latency and no noise events. Figure 1a shows an example log-intensity signal for a single pixel, with an overlay of binary ON and OFF events where pixel voltage crosses said threshold. Specifically, for small thresholds and limited bandwidth signals, the relation between event *k* and the log-intensity derivative can be approximated by Equation 1:


$$\begin{array}{l} L_{k}^{\prime }\approx \frac{\log\left(I_{k}\right)-\log\left(I_{k-1}\right)}{\Delta t_{k}}\equiv \frac{L_{k}-L_{k-1}}{\Delta t_{k}}, \end{array}$$ (1)

where *I*_*k*_ is the signal intensity at the time of the event, and Δ*t*_*k*_ is the time difference between the two, also called the inter-spike intervals. Throughout the text, we use the dash (′) to mark *temporal* derivatives of various functions. We note the refractory period, *t*_*rf*_, causes the log-intensity gap between events to be essentially different from the set θ_*ON*_ and θ_*OFF*_, with threshold crossing related to a reference level set *after* the refractory period is over. As shown in Figure 1b, relating the derivative to the contrast threshold can be approximated by a linear interpolation as in Equation 2:


$$\begin{array}{l} L_{k}^{\prime }\approx \frac{\theta \left(p_{k}\right)}{\Delta t_{k}-t_{rf}} \end{array}$$ (2)

$$\begin{array}{l} L_{k}\approx L_{k-1}+L_{k}^{\prime }\Delta t_{k}\approx L_{k-1}+\frac{\theta \left(p_{k}\right)\Delta t_{k}}{\Delta t_{k}-t_{rf}}\equiv L_{k-1}+{\tilde{\theta }}_{k} \end{array}$$

where θ(*p*_*k*_) represents either θ_*ON*_ or θ_*OFF*_ according to event polarity value, and ${\tilde{\theta }}_{k}$ is a corrected contrast value accounting for the refractory time (McHarg et al., 2022). This approximation holds only for event couples with identical polarity (ON after ON, OFF after OFF), and extending this notion to events with polarity flipping will require additional considerations, such as pixel event history and signal bandwidth. As a first-order approximation, we consider the values over the last two events, and generalize the corrected contrast to be a function θ(*p*_*k*−1_, *p*_*k*_) to obtain a better estimate of $L_{k}^{\prime }$. Specifically, we add a simple multiplier for polarity-flipping events. However, this provides only limited improvement to the estimated value, and additional spline-based methods can further improve this estimation (Fox et al., 2024). Last, using logarithmic differentiation, we found an estimate of the intensity derivative using Equation 3:


$$\begin{array}{l} L_{k}^{\prime }=\frac{I_{k}^{\prime }}{I_{k}}\to I_{k}^{\prime }=I_{k}L_{k}^{\prime }\approx \exp\left(L_{k-1}+{\tilde{\theta }}_{k}\right)\frac{\theta \left(p_{k-1},p_{k}\right)}{\Delta t_{k}-t_{rf}}. \end{array}$$ (3)

Here, all values are, in fact, *estimations* and written as true signal values only for convenience. This estimation becomes increasingly removed from the real signal as noise events (e.g., events unrelated to threshold crossing) and latency (the delay between threshold crossing and the reported timestamp) are introduced.

Expressing the per-event intensity derivative, $I_{k}^{\prime }$, holds several merits. First, as mentioned, it is possible to express the event stream as a linear system operator applied to the input scene (or its derivative). Second, we can use knowledge of either scene or system *dynamics* (such as active control of imaging optical parameters) to better infer scene information. Furthermore, as events represent timely *changes* in intensity, downstream computation that directly processes the magnitude of these changes can leverage the sparse, asynchronous nature of EVS. Specifically, we assign a *value* to each event related to the rate change in photon flux, thereby aiding model-based sensor designs and the development of physics-informed processing methods.

### 2.2 Single pixel signal estimation

Considering Equation 3, we perform a rough evaluation of the quality of this reconstruction, and its characteristics. This depiction represents many first-order approximations, while full statistical modeling of event inter-spike intervals (Hendrickson and Haefner, 2025; Graca and Delbruck, 2025) and interpolation methods for high-bandwidth signals are required for accurate interpolation-based reconstruction.

**Figure 2:**
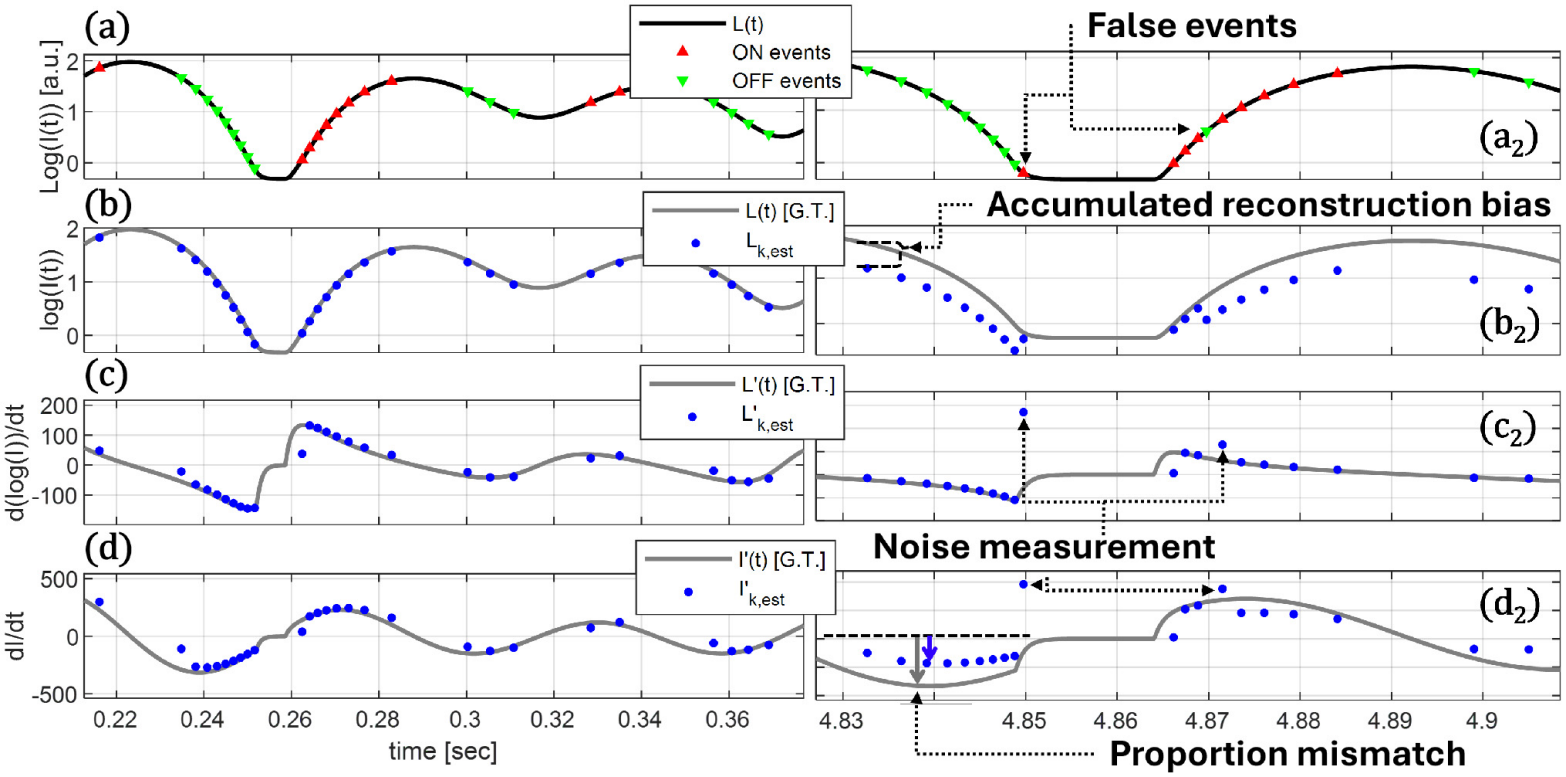
Pixel signal inference illustration. **(a)** A reference signal is measured by reporting ON and OFF events for each crossing of a corresponding threshold in its log-intensity profile. **(b)** A signal log-intensity estimation can be modelled by accumulating threshold crossing events according to the ON and OFF count. **(a**_**2**_**)** If event noise is introduced in the form of false events, **(b**_**2**_**)** reconstruction biases is prominent. **(c)** A first order estimate of the log-derivative is calculated by log-intensity difference, **(d)** and the estimate of each event's intensity derivative is calculated according to the product of the log-intensity derivative and intensity estimation. **(c**_**2**_**)** Here, noise events will lead to localised derivative miscalculations, **(d**_**2**_**)** and the reconstruction bias will lead to a proportional mismatch in the estimated derivatives.

An ideal event-pixel will report a threshold crossing without delay, then immediately reset to the signal value after the refractory period, and will not report any “noise” events. For a log-intensity signal with ideal event sampling, as shown in Figure 2a, we can reach a relatively good approximation of the original signal, as shown with blue dots in Figure 2b. The log-intensity derivatives, shown with blue dots in Figure 2c, and intensity derivatives, shown with a blue dot in Figure 2d, also mostly follow their ground truth signals except polarity flipping events highlighted in Figure 2b.

However, real event data will naturally include noise events of many shapes and forms, with limited examples presented in Figure 2a_2_. These accumulate to an intensity reconstruction error, exacerbated by the log-amplifying nature of EVS, as shown in Figure 2b_2_. The log-derivative estimation, as in Figure 2c_2_, is hardly affected by this process. This results in only a change in the proportion of the intensity derivative estimation for each event, shown in Figure 2d_2_ by comparing the blue dots (the derivative estimation) to the gray line ground truth signal. Specific noise events will produce corresponding noisy derivative measurements, but with limited downstream repercussions. While we introduce noise and latency to our simulation, and these are of course present in EVS recorded data, a full treatment of the effect of noise, together with how latency affects derivative reconstruction, is out of scope of this study.

### 2.3 Dynamic linear system for event data

A core aspect of CNI is defining the forward operator between a scene and the event data. As with conventional sensing, we may approximate the optical operator as a shift-invariant linear system when possible, but must account for the system's dynamics to fully appreciate the EVS measurement data. Figure 1c depicts the relation between a scene *U*, a dynamic shift-invariant linear optical system expressed as *T*, and the resulting projection *I*. For a system with such a *T* function, we may express it as a 2D matrix and formulate the system projection onto the sensor by *I* = T*_s_*U*, where *_s_ is the 2D-*spatial* convolution operation. The dynamics of this projection will follow Equation 4:


$$\begin{array}{l} I^{\prime }=\frac{\partial \left(T{*}_{s}U\right)}{\partial t}=\frac{\partial T}{\partial t}{*}_{s}U+T{*}_{s}\frac{\partial U}{\partial t}. \end{array}$$ (4)

For simplicity, we consider only static scenes with a dynamic projection system, such that $I^{\prime }=T^{\prime }{*}_{s}U$, and the general case will be considered in the Discussion section. If considering direct raw deconvolution of a scene, we may reconstruct it from the event-stream by expressing the inverse filter of the system using Equation 5:


$$\begin{array}{l} U_{est}=F_{i}\left\{\frac{F\left\{I^{\prime }\right\}}{F\left\{T^{\prime }\right\}}\right\}=F_{i}\left\{\frac{\hat{I^{\prime }}\left(\bar{\text{k}}\right)}{\hat{T^{\prime }}\left(\bar{\text{k}}\right)}\right\}, \end{array}$$ (5)

where F represents a Fourier transform and $F_{i}$ the corresponding inverse Fourier transform, and $\hat{I^{\prime }}\left(\bar{\text{k}}\right)$ and $\hat{T^{\prime }}\left(\bar{\text{k}}\right)$ represent the derivative image and optical transfer function in the frequency space $\bar{\text{k}}$. This method is limited to frequencies within a finite range where $\hat{T^{\prime }}\left(\bar{\text{k}}\right)>0$, to avoid isolated poles, and neglects the effect of noise amplified by deconvolution. To address these limitations, we instead choose the Wiener deconvolution (Joseph, 1968)


$$\begin{array}{l} U_{est}=F_{i}\left\{\frac{{\hat{T^{\prime }}}^{*}\left(\bar{\text{k}}\right)\hat{I^{\prime }}\left(\bar{\text{k}}\right)}{|\hat{T^{\prime }}\left(\bar{\text{k}}\right){|}^{2}+\lambda \left(\bar{\text{k}}\right)}\right\}, \end{array}$$ (6)

In Equation 6 ${\hat{T^{\prime }}}^{*}\left(\bar{\text{k}}\right)$ is the complex conjugate of the deconvolution filter, and $\lambda \left(\bar{\text{k}}\right)$ is a heuristic regularization, and can be replaced by the noise mean spectral density when it is known. Here we use a frequency dependent function of $\lambda \left({\text{k}}_{r}\right)=\eta_{1}+\eta_{2}{\text{k}}_{r}^{2}$, where η_1_ = 2 and $\eta_{2}=1.2\times 10^{-10}\left[m^{2}/rad^{2}\right]$ are manually chosen.

Inverse filters in the context of image reconstruction are conventionally performed on dense 2D matrices. We too employ a 2D Fast Fourier Transform (FFT) to analyze both event derivatives and *T*′, as a placeholder for event-native Fourier transforms that will be explored in future study (Sabatier et al., 2017; Tapia et al., 2024). A frame is constructed choosing only the most recent derivative value per pixel, multiplied by a decay factor (accounting for the time since that event was reported) before performing a 2D FFT on the image.

As an example, consider imaging a point-source by a dynamically varying focal length system as in Figure 1c. Specifically, consider a system similar to that of Ralph et al. (2024), where a stationary star field (neglecting atmospheric turbulence) is imaged using a telescope fixed liquid lens. Modulating the focal length around the sensor focal point enabled improved noise rejection, star-brightness estimation, and the avoidance of the tedious chore of focusing an event camera. For now, however, we consider only an equivalent system with the thin-lens approximation, monochromatic point sources, a narrow field of view, and no additional aberrations. For such a system, the projection of a point-source will be a uniformly expanding and contracting spot with a constant overall intensity as shown in Figures 1d, e, where the transfer function and its derivatives can be analytically described (see Supplementary material).

### 2.4 Spatial-temporal kernels

**Figure 3:**
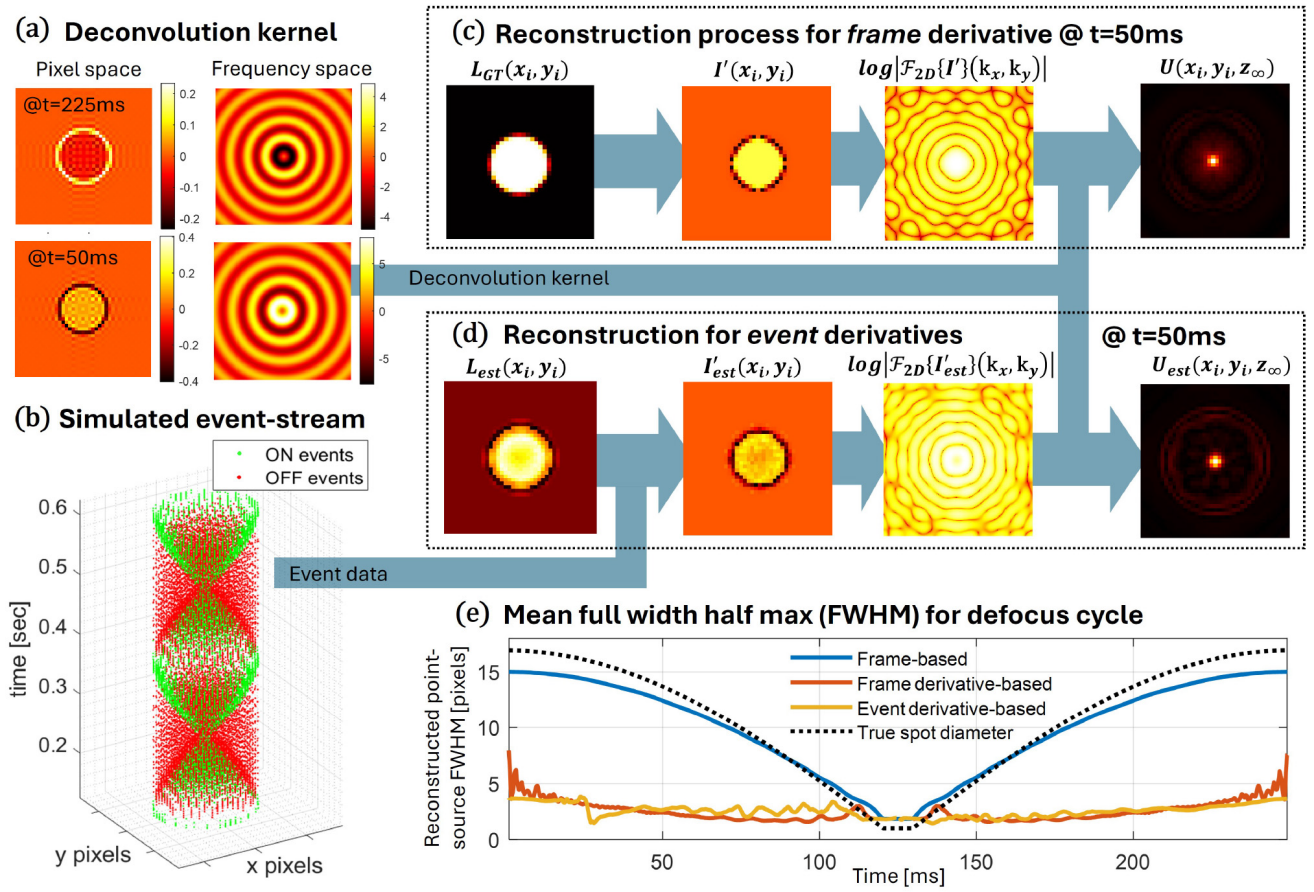
Estimating point-source via deconvolution of event streams. **(a)** A dynamic deconvolution kernel for a tunable lens system is defined analytically and presented here, in both pixel space on the left and the frequency domain on the right, for two time points. The top row presents a moment in the expansion phase and the bottom row a moment in mid-contraction. **(b)** A simulation generates an event stream expected for a point source through a modulated de-focus optical model. **(c)** The original frame used to simulate the event stream is also used to benchmark event-based processing. Here we present, from left to right, an example of such a processing sequence: the original frame, its derivative, the derivative's 2D Fourier transform, and the resulting Wiener deconvolution point-source reconstruction using the contemporaneous kernel. **(d)** A similar process on the simulated event data shows, from left to right, the estimated log-intensity frame, event-based derivative estimation, its 2D Fourier transform, and the resulting point-source reconstruction. **(e)** For each moment in the defocus modulation cycle, the Full Width Half Max of the reconstructed point-source is shown for: the original frame (blue), the frame-based derivative (red), and the event-based derivative (yellow), alongside the true projected spot diameter (dotted line).

We considered the expression $\hat{T^{\prime }}\left(\bar{\text{k}}\right)$ in Equations 5, 6, dubbed here the deconvolution kernel. This can be calculated analytically from a projection model, estimated via simulation, measured using an impulse-response source, or jointly estimated with the event data using model-based optimization methods. For our tunable focal-length thin lens system, under the assumptions of shift invariance and linearity, we can analytically compute the PSF as a function of time (see Supplementary material). Classically, a PSF describes the intensity distribution of a point-source illumination on the sensor plane. Using Equation 7 we consider the temporal derivative of this PSF as the deconvolution kernel:


$$\begin{array}{l} {\hat{T}}^{\prime }\left({\text{k}}_{r},t\right)=2\pi R\left(t\right)\cdot \left(g\left(t\right)\frac{J_{1}\left(R{\text{k}}_{r}\right)}{{\text{k}}_{r}}+h\left(t\right)J_{0}\left(R{\text{k}}_{r}\right)\right), \end{array}$$ (7)

where *R*(*t*) represents the momentary spot radius (a function of the effective focal length and other system parameters), *J*_*n*_(*x*) is the Bessel function of order *n*, and **k**_*r*_ is the radial frequency, translated to sensor's frequency domain by ${\text{k}}_{r}=\sqrt{{\text{k}}_{x}^{2}+{\text{k}}_{y}^{2}}$. Here, *g*(*t*) and *h*(*t*) denote the inter-spot intensity profile and the outer-ring impulse function, respectively (these are evolving functions that represent changes in spot shape and are detailed in the Supplementary material). Figure 3a shows the images both in frequency space and in the spatial domain on a 2D pixel matrix at different points in time in expansion and contraction phases. The spatial-domain images show the expected outer ring with high derivative values (representing the transition from spot illumination to background illumination, or vice versa), and the internal derivative values representing an increase (or decrease) as spots concentrate illumination on fewer pixels.

However, not all systems can be modeled as easily. Combined event and optical simulators (Hu et al., 2021; Joubert et al., 2021; Kruger et al., 2025) to estimate the deconvolution kernel, which presents a useful middle ground. While generalized event simulators are not yet mature or computationally efficient, their optical counterpart are. This allows the development of first-order deconvolution kernels based on a system's design.

We also commented on an additional avenue for implementing spatiotemporal priors in our framework, introduced even earlier in the computation. When accumulating the intensity estimation map according to Equation 1, one might choose to expand the pixel-level estimate to account for the local intensity map and to introduce scene- and pixel-level priors. To clarify this notion by way of example, we often encounter hyper-sensitive pixels (or even “hot pixels” that fire regardless of changes in photon rate) in EVS data. While these are often treated by a pre-processing filter stage, we implemented a different approach. We dictate accumulated intensity estimation, *I*(*x*_*k*_, *y*_*k*_), to be mostly contributed by *neighboring* pixel events, rather than solely from the events it reports. If no such events arrive, the intensity estimation hardly changes, and the derivative estimation remains small. However, if neighboring pixels fire just as often (as will happen in most optical systems with true illumination changes), the local pixel-cluster intensity estimate will rise in unison. Here, when an event occurs at (*x*_*k*_, *y*_*k*_) we add exp(*L*_*k*_)*ker_*NN*_ to the intensity map centered at (*x*_*k*_, *y*_*k*_), as described by Equation 8:


$$\begin{array}{l} ker_{NN}\equiv \left[\begin{array}{ccc} 0.065 & 0.178 & 0.065\\ 0.178 & 0.024 & 0.178\\ 0.065 & 0.178 & 0.065 \end{array}\right]. \end{array}$$ (8)

We applied this convolutional smoothing kernel to the estimated log-intensity update image, calculated according to Equation 2, on both simulated data and real-world EVS data to improve stability in the presence of random noise events.

### 2.5 Scene estimation algorithm

Combining the per-event intensity derivative reconstruction and the Wiener deconvolution for point-source estimation using the spatial-temporal kernels described above can be performed on a per-event basis. In this approach, each event contributes information relevant to the desired signal and need not be considered further in a full-frame context. However, in this demonstration, we project the events onto a frame before performing a 2D FFT as a placeholder for future event-native solutions. This avenue is chosen because current event-based FFT methods (Sabatier et al., 2017; Tapia et al., 2024) support only spatial *or* temporal Fourier analysis and are not as computationally efficient as conventional 2D FFT.

We thus estimated the sensor intensity derivative frame, $I_{est}^{\prime }\left(x_{i},y_{i}\right)$, at uniformly separated time samples *t*, similar to the description above, with several differences. (a) We accumulated events within a time step to update the intensity estimation, using a continuous-time filter as used in Scheerlinck et al. (2019), and starting with an initial guess. In the simulation, we base the initial guess on the known input to the event simulator, or just assume uniform intensity (no sources) for EVS recording onset. (b) The image derivative is updated using only the most recent events for each pixel within a time step, and an additional decay factor of exp(−(*t* − *t*_*k*_)/τ). The need for this filter stems from the projection to a 2D image frame and the need to “relax” derivative values that have not been updated with recent events. (c) For each time-step we perform a 2D FFT operation on the derivative image $I_{est}^{\prime }\left(x_{i},y_{i}\right)$ to get $\hat{I^{\prime }}\left({\text{k}}_{x},{\text{k}}_{y}\right)$. Zero-padding of the original image was used to smooth the frequency-space images.

## 3 Results

### 3.1 Deconvolution of simulated data

Before processing the event stream, we run our deconvolution kernel on the derivative of the expected image intensity as depicted in Figure 3c. The process is similar to the algorithm steps above, but without estimating frames or derivatives, since these are computed directly from our optical-modulated focal-length model and its temporal derivative. The image frames of the expanding and contracting spot follow a sinusoidally focal-length-modulated tunable lens model, calculated in an upsampled pixel space to obtain smooth spatiotemporal changes. Optical parameters of the model are chosen to be similar to those of our liquid lens used for telescope imaging of a star field. The resulting 2D FFT of the “ground truth” derivative is then used in the same Wiener deconvolution, as in Equation 6, to reconstruct the point-source at infinity.

As proof of concept of this method for event-based imaging, we input the images of the optical model to an event-stream simulation using Kruger et al. (2025), and an event-stream example is presented in Figure 3b. Simulation time steps are chosen to be on the order of several microseconds to prevent event-batching, and pixel parameters were mostly chosen to fit the EVS Prophesee Gen4 device values (pixel size, dark and source follower time constants, and dark-current), apart from non-uniformity across pixel contrast threshold values set to 0.2 ± 0.02 and the omission of any noise events—both chosen specifically for this stage of framework development. Notably, uniform contrast thresholds across all pixels led to symmetries in the event projections, creating misleading, albeit beautiful, frequency images.

Following the interpolation of events, as described in Section 2.5, we process the event-stream to produce the full point-source reconstruction. In Figure 3d, we see from left to right the log-intensity estimation, the intensity derivative estimation using the latest batch of events, the Fourier image of the event-based derivative, and finally the point-source reconstruction using the contemporary deconvolution kernel.

We calculate the Full Width Half Max (FWHM) of the four spots involved: the original spot projected on the image plane, the attempt to reconstruct the point-source from that image, a frame-derivative reconstruction, and the event-based derivative reconstruction. These are presented in Figure 3e within a full cycle of the lens focal sweep. The event-based reconstruction is compared with the original spot and the frame-derivative “ground truth” calculation, and we clearly see both validation of the method for reconstructing the point source and performance comparable to that of the ideal scenario. We note that the FWHM metric choice is but one amongst many, and future studies should consider additional aspects such as energy loss to side lobes, and intensity estimation accuracy. We also note that the attempt to calculate the deconvolution using only the original frame-based spot fails miserably, as shown by the blue line in Figure 3e—and using the correct kernel, of course. The reason for that is that the flat featureless spot contains many zeros in the frequency domain, making deconvolution methods a poor choice for point-source reconstruction for such PSFs.

In this example, we presented representative results of source estimation, while a full video of these images across multiple cycles of focal-length modulation is provided as Supplementary material. As expected, during periods with limited dynamics (when the spot is at either the maximum or the minimum and the dynamic optical transfer function becomes a null operator) and many polarity flips, the source estimation diverges in both shape and intensity. This suggests that the signal noise floor in these dynamic signals needs to be carefully accounted for in both sensor dynamics and scene dynamics (Kruger et al., 2025).

**Figure 4:**
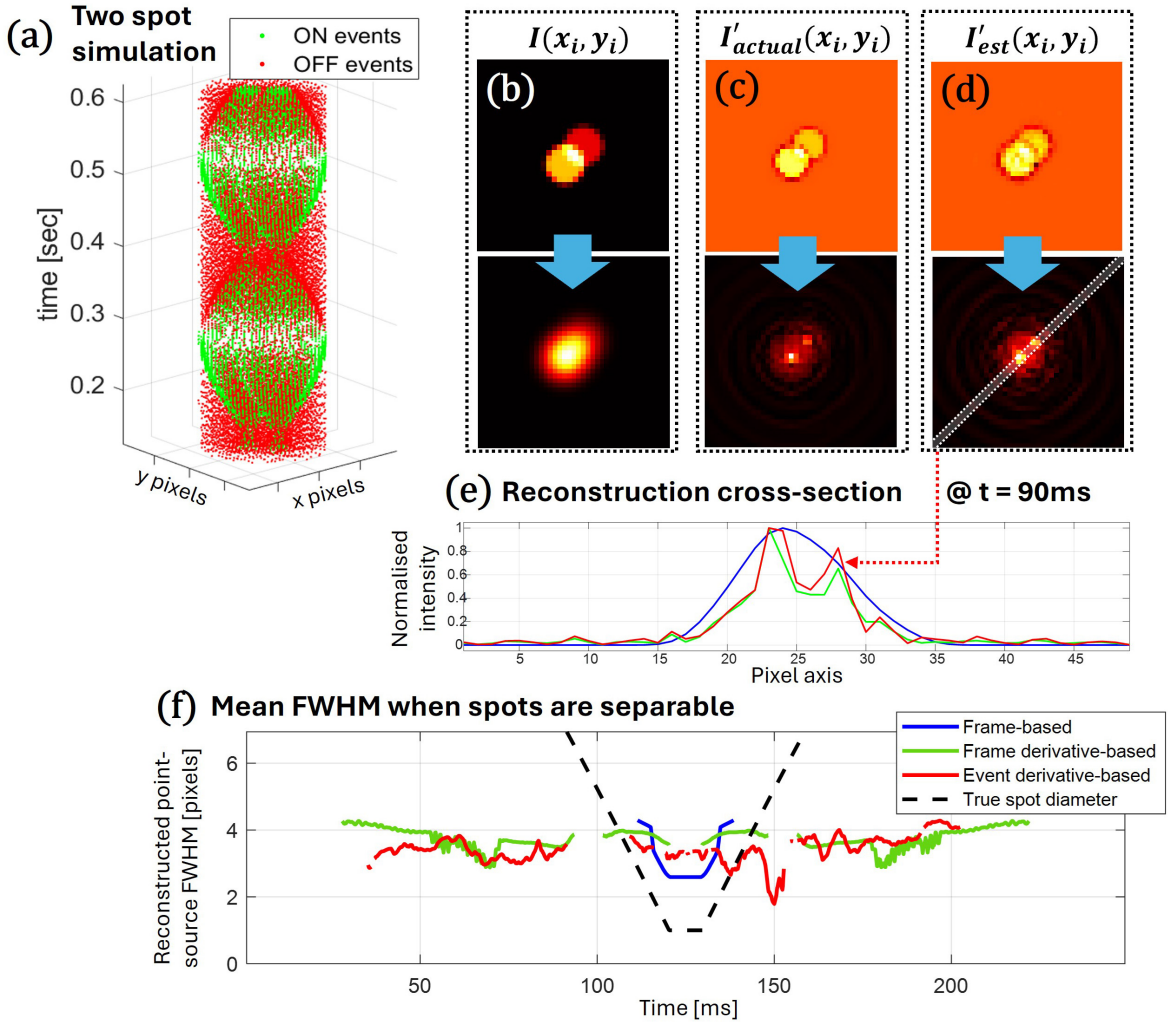
Deconvolution of multiple sources. **(a)** A simulated event-stream of two point-sources, one with double the brightness relative to the other, and distanced by 5 pixels. The deconvolution process for **(b)** the original spot intensity frame, **(c)** the frame-derivative intensity, and **(d)** the event-based derivative estimation is presented with the top row showing the data source, and the bottom showing the resulting point-source estimation. **(e)** A cross-section of the reconstruction image, along the line crossing both point-sources, is plotted for each reconstruction method for *t* = 90 ms—blue for frame-based, green for frame-derivative, and red for our event-based deconvolution. **(f)** When the two spots are separable in the resulting cross section, the mean FWHM (average of both spots and across several computational cycles) is presented as a function of time (250 ms modulation cycle). Similarly, the blue line represents frame-based estimation, the green line represents frame-derivative estimation, the red line represents event-derivative estimation, and the dotted line represents the diameter of the original simulated spot.

To further demonstrate the power of this tool, we test its performance on a source confusion problem using a dual-source simulated data stream. The out-of-focus imaging system will lead to overlapping events (events generated at pixels affected by both sources' intensities) over prolonged periods of lens modulation. We choose one source to be twice as intense as the other, and position the points 5.6 pixels apart on the diagonal of the sensor plane. In Figure 4a, the event-stream for points positioned five pixels apart is portrayed. The deconvolution process is implemented as with the single spot, and we present example results for the various methods at a single point in time during spot contraction. The original spot deconvolution results are shown in Figure 4b, the frame derivative result in Figure 4c, and our event-based deconvolution in Figure 4d.

The cross section of each reconstruction image, taken along the diagonal where both sources are positioned, is presented in Figure 4e, where comparable performance of both derivative-based methods is evident and both are superior to an attempt to perform deconvolution on the original spot image. We use the Rayleigh criteria to ascertain successful source separation (pixel value mid-point between two peaks a factor of 0.8 of the lowest peak) throughout the computational cycle, and measure the overall mean FWHM for each across all modulation cycles—as shown in Figure 4f. Only regions of consistent point separation across all cycles are presented. While frame-derivative and event-based FWHM results are comparable, the event-based method falls short in two respects. First, we observed less reliable separation, with more gaps indicating unsuccessful source confusion, even when the reference frame-derivative method succeeded. Second, the event-derivative result shows larger variance in resulting FWHM. Aspects not presented here, but clearly evident in the video clip attached to the Supplementary material representing the full reconstruction image as a function of time, are the higher leakage of energy to side lobes, and failure points around the polarity flipping event (as expected from the description in Section 2.2).

Another aspect to note is the intensity ratio between the reconstructed point sources, as these were modeled with a factor of 0.5 between the top-right and bottom-left spots in the image. The current reconstruction does not accurately capture this ratio, but the energy is typically distributed, with a preference for the higher-intensity source. As mentioned, the FWHM is only one metric of many others that require dedicated treatment as this framework is developed.

### 3.2 Synthetic deconvolution kernel for measured event data

In this section, we test the method on data collected in Ralph et al. (2024), where events are generated by modulating the focal length of a 2,800-mm telescope imaging the star field “M47” (Messier 47/NGC 2442). The telescope was equipped with an Optotune El-10-30-C-VIS liquid lens system and modulated to produce a sinusoidal variation in focal length, thereby dynamically projecting the star field as in the optical model described above. Application of the deconvolution method quickly reveals deviations from this model, foremost in the imaging of a broad-spectrum image through a liquid lens system, which is hard-pressed by the thin-lens approximation. For instance, slight spherical aberrations will result in a highly non-uniform PSF when out of focus, thereby breaking the symmetry assumed for focusing before and after the sensor. A realistic PSF, possibly derived using an optical simulator, would provide a spot with more energy on the periphery when focused behind the detector, and a central-heavy energy distribution when focused before the detector. Furthermore, dynamic behavior that is unrelated to the focal length modulation is present—namely, atmospheric turbulence. While these are interesting to consider, we would need to include methods to validate any observed wavefront shifts resulting from such effects.

**Figure 5:**
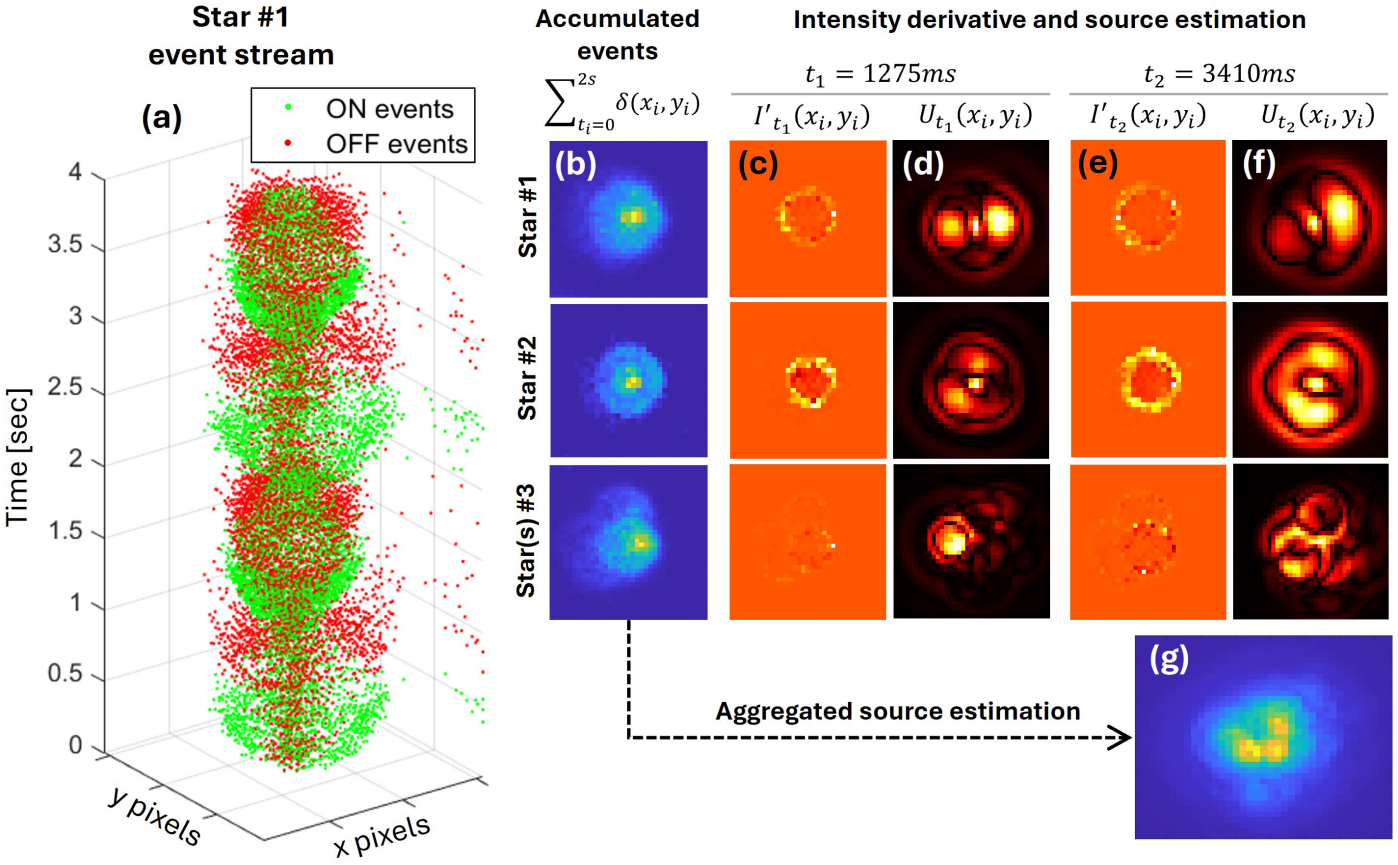
Sample source estimation from measured EVS data on modulated focal length images of a star field. **(a)** Example event stream of an image of a star acquired with 0.5 Hz focal length modulation. Deconvolution from events is applied on three different regions, with depiction of **(b)** accumulated raw events, and the calculated **(c, e)** intensity derivative from events and **(d, f)** the source estimations, each for two points in time. **(g)** The average normalized source estimation, strategically accumulated over three cycles (using only the rapid expansion and contraction periods of the modulation cycle), is presented for a region containing several stars in close proximity.

Nonetheless, for extended periods within the processing cycle, we can obtain a clear point-source inference, with additional energy diverted to its periphery, representing various scene dynamics and residual optical features. Using data from the M47 star field, we consider three sources and perform deconvolution of event data from their vicinity. We choose data from the lens modulation frequency of 0.5 Hz, having a maximal number of events per cycle, and a sample event stream for the first two cycles of source #1 is presented in Figure 5a. The regions noted as star#1 and star#2 contain only a single notable source (designations HD 37042 and HD 37062, respectively), whereas the region of star(s)#3 is, in fact, a cluster of three bright stars (HD 37020, HD 37022, and HD 37023). In Figures 5b–f, we show the processing results for each of these sources: star#1, star#2, and star #3, presented from top to bottom. Figure 5b presents the accumulated events over a period of 2 s, the columns of Figures 5c, e show the estimated event-based derivative calculated for *t*_1_ = 1, 275*ms* and *t*_2_ = 3, 410*ms* respectively, while the columns of Figures 5d, f present the resulting source reconstruction for these same images. Both cases are chosen so that the focus point is behind the sensor plane (where the PSF is similar to the thin-lens model PSF). Considering the overall effect of this process, we strategically accumulate normalized source-estimation results over several cycles. Based on the insights gained in the simulation stage regarding optimal FWHM values, we collect reconstruction images only during the rapid expansion or contraction phases of the PSF. Also, our simplistic model for calculating the deconvolution kernel is better aligned with the actual optical projection when the focus is behind the sensor plane. As shown in Figure 5g, the deconvolution provides a sharper point-source localization compared to just raw events accumulation in the case of multiple sources. However, a direct comparison to real star data has yet to be made. A full video stream of the processed EVS data is available in the Supplementary material.

## 4 Discussion

In this study, we proposed a new framework for EVS data processing, aimed at initiating a discussion of a generalized physics-informed imaging approach for CNI applications. These typically have an optical projection design that doesn't follow the intuitive pinhole camera model or includes a form of engineered dynamics. Our example for scene reconstruction from a modulated focal-length system using derivative estimation leaves ample room for improvement with issues related to polarity switching, accuracy around drastic intensity jumps, and handling non-ideal pixel characteristics (such as light-dependent latency, threshold non-uniformity, and detailed noise estimation). Furthermore, quantifying the contribution of frame initialization, either through external frame data or initial guesses, to reconstruction accuracy has not been performed.

As EVS sensor models develop, so will the underlying assumptions used for event interpolation, leading to better intensity and intensity derivative estimations. Interpolation methods using more than the last two events can further this goal, or other successful intensity-from-events reconstruction methods (Kim et al., 2014; Munda et al., 2018; Scheerlinck et al., 2019; Fox et al., 2024; Antil and Sayre, 2023) may be adapted to estimate intensity derivatives as well. In this context, the various types of EVS noise modes will affect reconstruction accuracy, and this effect must be fully quantified. The ability of our event-based derivative reconstruction to directly inform a physics-related quantity (the pixel-level change in photon flux) will be beneficial in defining such meaningful performance metrics.

The use of simulation data and analytical deconvolution kernels in this work provided insight into performance aspects, namely the ability to closely match FWHM values calculated without events. However, applications that use real EVS data still require closer attention to this method's failure modes. For instance, a measured impulse response or PSF could better capture both system dynamics and the sensor's stochastic nature, but would require dedicated measurement and calibration procedures. We note, for instance, that the interpolation method relies heavily on knowledge of the contrast threshold, and calibration procedures for pixel non-uniformity (Wang et al., 2020; McReynolds et al., 2023) may also prove important.

The example in this study considers the operator of only a dynamic kernel on a static scene. Handling a system with both scene *and* operator dynamics, however, requires inference of both static and dynamic scenes. One option might be to compute separate slow and fast dynamics, assuming a quasi-static scene or a quasi-static operator, and perform inference on different time scales. Alternatively, solving the inverse problem for both the static and dynamic scene outputs simultaneously can be done using the many data-driven methods for such overdetermined problems.

We mentioned using frame-based accumulation as a placeholder to enable quick use of conventional 2D-FFT tools, while eroding the benefits of the sparse asynchronous event representation. Replacing these with event-based Fourier transforms, or Non-Uniform Fast Fourier Transform (NUFFT), may improve performance and inspire applications where image reconstruction is not the goal.

The example closing the results section, with Figure 5 showing the static sources' projection onto the sensor, which is affected by both the imaging system and atmospheric dynamics, suggests the possibility of simultaneous event-based decoding of both the scene and the optical system. Thus, layers of flexibility may be added to EVS systems, including control of system parameters (such as spatial bandwidth, spectral encoding, etc.), separation of the scene's persistent and dynamic characteristics, and detection of fast features of interest at rates surpassing those of any conventional frame-based sensor.

## Figures and Tables

**Table:** 

Symbol	Definition
Sensor and signal variables	
*I*	Scene intensity (photon flux)
*L*	Log-intensity, *L* ≡ log(*I*)
*L*′	Temporal derivative of log-intensity, d*L*/d*t*
*I*′	Temporal derivative of intensity, d*I*/d*t*
*e*_*k*_	Event tuple (*x*_*k*_, *t*_*k*_, *p*_*k*_) for the *k*-th event
*x*_*k*_	Pixel location (*x*_*k*_, *y*_*k*_) of the *k*-th event
*t*_*k*_	Timestamp of the *k*-th event
*p*_*k*_	Polarity of the *k*-th event (+1 for ON, −1 for OFF)
Δ*t*_*k*_	Inter-spike interval between events *k* − 1 and *k*
*L*_*k*_	Estimated log-intensity at event *k*
$L_{k}^{\prime }$	Estimated log-intensity derivative at event *k*
$I_{k}^{\prime }$	Estimated intensity derivative at event *k*
Sensor model parameters	
θ_ON_	Log-intensity threshold for ON events
θ_OFF_	Log-intensity threshold for OFF events
θ(*p*_*k*_)	Threshold value selected by event polarity
θ(*p*_*k*−1_, *p*_*k*_)	Corrected contrast threshold accounting for polarity flipping
${\tilde{\theta }}_{k}$	Refractory-period-corrected contrast value
*t*_rf_	Refractory period duration
Optical system variables	
*U*	Input scene field (complex wavefront or intensity)
*T*	Shift-invariant linear optical transfer operator
*T*(*t*)	Time-varying optical transfer operator
*T*′	Temporal derivative of the optical transfer operator
${\hat{T}}^{\prime }\left(\bar{k}\right)$	Fourier-domain representation of *T*′
$\bar{k}$	2D spatial frequency vector (*k*_*x*_, *k*_*y*_)
*k*_*r*_	Radial spatial frequency, $k_{r}=\sqrt{k_{x}^{2}+k_{y}^{2}}$
Point spread function and deconvolution	
*R*(*t*)	Momentary defocus spot radius
*J*_*n*_(*x*)	Bessel function of the first kind, order *n*
*g*(*t*)	Inter-spot intensity profile coefficient
*h*(*t*)	Outer-ring impulse coefficient
$I_{t}^{\prime }\left(x_{i},y_{i}\right)$	Estimated intensity derivative frame at time *t*
${Î}^{\prime }\left(\bar{k}\right)$	Fourier-domain derivative image
*U*_est_	Estimated scene (point-source reconstruction)
$\lambda \left(\bar{k}\right)$	Wiener deconvolution regularization function
τ	Decay time constant for derivative frame relaxation

## Data Availability

The datasets presented in this study can be found in online repositories. The names of the repository/repositories and accession number(s) can be found in the article/Supplementary material.

## References

[B1] Antil H. Sayre D. (2023). Bilevel inverse problems in neuromorphic imaging. Inverse Probl. 39:094003. doi: 10.1088/1361-6420/ace7c7

[B2] Arja S. Kruger N. Marcireau A. Ralph N. O. Afshar S. Cohen G. . (2025). Seeing like a cephalopod: colour vision with a monochrome event camera. arXiv [preprint]. arXiv:2504.10984. doi: 10.48550/arXiv.2504.10984

[B3] Baird R. G. Majumder A. Menon R. (2025). Dynamic spectral fluorescence microscopy via event-based & CMOS image-sensor fusion. Opt. Express 33, 2169–2178. doi: 10.1364/OE.545667 39876372

[B4] Bian L. Chang X. Xu H. Zhang J. (2024). Ultra-fast light-field microscopy with event detection. Light: Sci. Appl. 13:306. doi: 10.1038/s41377-024-01603-1 39511142 PMC11544014

[B5] Cai M. Galor D. Kohli A. P. S. Yates J. L. Waller L. (2025). Event2Audio: event-based optical vibration sensing. arXiv [preprint]. arXiv:2507.03273. doi: 10.48550/arXiv.2507.03273

[B6] Fox G. Pan X. Tewari A. Elgharib M. Theobalt C. (2024). “Unsupervised event-based video reconstruction,” in 2024 IEEE/CVF winter conference on applications of computer vision (WACV) (Waikoloa, HI: IEEE), 4167–4176. doi: 10.1109/WACV57701.2024.00413

[B7] Graca R. Delbruck T. (2025). Towards a physically realistic computationally efficient DVS pixel model. arXiv [preprint]. arXiv:2505.07386.

[B8] Habuchi S. Takahashi K. Tsutake C. Fujii T. Nagahara H. (2024). Time-efficient light-field acquisition using coded aperture and events. arXiv [preprint]. arXiv:2403.07244. doi: 10.48550/arXiv.2403.07244

[B9] Hendrickson A. J. Haefner D. P. (2025). Beyond discretization: a continuous-time framework for event generation in neuromorphic pixels. arXiv [preprint]. arXiv:2504.02803.

[B10] Hu Y. Liu S.-C. Delbruck T. (2021). “v2e: from video frames to realistic DVS events,” in 2021 IEEE/CVF conference on computer vision and pattern recognition workshops (CVPRW), 1312–1321. doi: 10.1109/CVPRW53098.2021.00144

[B11] Joseph W. G. (1968). Introduction to Fourier Optics. Columbus, OH: McGraw-Hill Inc.

[B12] Joubert D. Marcireau A. Ralph N. Jolley A. van Schaik A. Cohen G. . (2021). Event camera simulator improvements via characterized parameters. Front. Neurosci. 15:702765. doi: 10.3389/fnins.2021.702765 34385903 PMC8353146

[B13] Kim H. Handa A. Benosman R. Ieng S.-H. Davison A. (2014). “Simultaneous mosaicing and tracking with an event camera,” in Proceedings of the British machine vision conference 2014 (Nottingham: British Machine Vision Association), 26.1–26.12. doi: 10.5244/C.28.26

[B14] Kong F. Lambert A. Joubert D. Cohen G. (2020). Shack-Hartmann wavefront sensing using spatial-temporal data from an event-based image sensor. Opt. Express 28:36159. doi: 10.1364/OE.409682 33379717

[B15] Kruger N. Arja S. Andrew E. Monk T. Schaik A. V. (2025). “Performance metrics for neuromorphic imaging,” in Quantum sensing and nano electronics and photonics XXI (San Francisco: SPIE), Vol. 13376, 74–82. doi: 10.1117/12.3041873

[B16] McHarg M. G. Balthazor R. L. McReynolds B. J. Howe D. H. Maloney C. J. O'Keefe D. . (2022). Falcon Neuro: an event-based sensor on the International Space Station. Opt. Eng. 61:085105. doi: 10.1117/1.OE.61.8.085105

[B17] McReynolds B. Graca R. Delbruck T. (2023). Exploiting alternating DVS shot noise event pair statistics to reduce background activity. arXiv [preprint]. arXiv:2304.03494.

[B18] Munda G. Reinbacher C. Pock T. (2018). Real-time intensity-image reconstruction for event cameras using manifold regularisation. Int. J. Comput. Vis. 126, 1381–1393. doi: 10.1007/s11263-018-1106-2

[B19] Qu Z. Zou Z. Boominathan V. Chakravarthula P. Pediredla A. (2024). Event fields: Capturing light fields at high speed, resolution, and dynamic range. arXiv [preprint]. arXiv:2412.06191. doi: 10.48550/arXiv.2412.06191

[B20] Ralph N. O. Maybour D. Marcireau A. Dennler N. Arja S. Kruger N. . (2024). Active neuromorphic space imaging and focusing using liquid lenses. TechrXiv. doi: 10.36227/techrxiv.173152542.28624013/v1

[B21] Sabatier Q. Ieng S.-H. Benosman R. (2017). Asynchronous event-based fourier analysis. IEEE Trans. Image Process. 26, 2192–2202. doi: 10.1109/TIP.2017.2661702 28186889

[B22] Scheerlinck C. Barnes N. Mahony R. (2019). “Continuous-time intensity estimation using event cameras,” in Computer Vision – *ACCV 2018*, eds. C. Jawahar, H. Li, G. Mori, and K. Schindler (Cham: Springer International Publishing), Vol. 11365, 308–324. Series Title: Lecture Notes in Computer Science. doi: 10.1007/978-3-030-20873-8_20

[B23] Shah S. Chan M. A. Cai H. Chen J. Kulshrestha S. Singh C. D. . (2024). CodedEvents: optimal point-spread-function engineering for 3D-tracking with event cameras. arXiv [preprint]. arXiv:2406.09409 [cs]. doi: 10.1109/CVPR52733.2024.02387

[B24] Tapia R. Dios J. R. M.-d. Ollero A. (2024). eFFT: an event-based method for the efficient computation of exact fourier transforms. in IEEE Trans. pattern Anal. Mach. Intell. 46, 9630–9647. doi: 10.1109/TPAMI.2024.3422209 38954586

[B25] Wang Z. Ng Y. Goor P. V. Mahony R. (2020). Event camera calibration of per-pixel biased contrast threshold. arXiv [preprint]. arXiv:2012.09378.

[B26] You Y. Wang Y. Cai Y. Zhu M. He B. (2025). 3D localization using lensless event sensors for fast-moving objects. Digit. Signal Process. 161:105077. doi: 10.1016/j.dsp.2025.105077

[B27] Zhang Z. Yang H. Li J. Chong S. W. Eshraghian J. K. Yong K.-T. . (2025). Neuromorphic imaging cytometry on human blood cells. Neuromorphic Comput. Eng. 5:024001. doi: 10.1088/2634-4386/adc6b4

[B28] Zhang Z. Yezzi A. Gallego G. (2022). “Formulating event-based image reconstruction as a linear inverse problem with deep regularization using optical flow,” in IEEE transactions on pattern analysis and machine intelligence, 1–18. arXiv:2112.06242. 10.1109/TPAMI.2022.3230727 37015430

[B29] Zhu S. Ge Z. Wang C. Han J. Lam E. Y. (2024a). Efficient non-line-of-sight tracking with computational neuromorphic imaging. Opt. Lett. 49, 3584–3587. doi: 10.1364/OL.530066 38950215

[B30] Zhu S. Wang C. Liu H. Zhang P. Lam E. Y. (2024b). “Computational neuromorphic imaging: principles and applications,” in Computational optical imaging and artificial intelligence in biomedical sciences (San Francisco: SPIE), Vol. 12857, 4–10. doi: 10.1117/12.3012192

