# Supplementary material for: Optical linear systems framework for event sensing and computational neuromorphic imaging
Source: Front Neurosci. 2026 May 19;20:1831543. doi: 10.3389/fnins.2026.1831543 (PMC13227969; doi:10.3389/fnins.2026.1831543)
Supplement: Supplementary file 1 [file Data_Sheet_1.pdf]

# *Optical Linear Systems Framework for Event Sensing and Computational Neuromorphic Imaging - Supplementary Material*

## 1 ANALYTICAL DESCRIPTION OF THE DEFOCUS TRANSFER FUNCTION AND DECONVOLUTION KERNEL

This supplementary derives the dynamic (time-varying) PSF derivative and its Fourier-domain representation used to construct the deconvolution kernel for the ideal focal-length–modulated lens model. The derivation below follows the same assumptions and notation used in the accompanying notes.

### 1.1 Dynamic focal length

We model the effective focal length as a sinusoidal modulation

$$\begin{aligned} f(t) &= f_0 + \Delta f \sin(\omega_0 t), \\ f'(t) &= \Delta f\, \omega_0 \cos(\omega_0 t), \end{aligned} \tag{S1}$$

where $f_0$ is the base focal length, $\Delta f$ is the modulation amplitude, and $\omega_0$ is the modulation angular frequency.

### 1.2 Geometric defocus radius and its rate of change

Assume a circular aperture of diameter $A$, imaging a point source at infinity, with sensor–lens distance $d_2$. The pupil illumination is $I_0$, and $I_{\text{bg}}$ is a spatially uniform background level. Under the idealized "uniform disc" defocus model, the blur radius on the sensor plane is

$$R(t) = A \left| \frac{d_2}{f(t)} - 1 \right|. \tag{S2}$$

Differentiating yields

$$\begin{aligned} R'(t) &= -\text{sign}\left(\frac{d_2}{f(t)} - 1\right) A \frac{d_2 f'(t)}{f(t)^2} \\ &= -\text{sign}\left(\frac{d_2}{f(t)} - 1\right) A \frac{d_2 \Delta f\, \omega_0 \cos(\omega_0 t)}{(f_0 + \Delta f \sin(\omega_0 t))^2}. \end{aligned} \tag{S3}$$

determining rate of image-plane spot expansion ($R'(t) > 0$) or contraction ($R'(t) < 0$).

## 1.3 Intensity model and decomposition of the temporal derivative

The defocused PSF is modeled as a spatially uniform disc of radius $R(t)$ on top of a uniform background. Total illumination does not change with focus; therefore the local disc level scales with $1/R(t)^2$:

$$I(r,t) = \begin{cases} I_{\mathrm{bg}} + \dfrac{I_0}{\pi R(t)^2}, & r < R(t), \\ I_{\mathrm{bg}}, & \text{otherwise}, \end{cases} \tag{S4}$$

or equivalently using a radial unit-step $U(\cdot)$,

$$I(r,t) = I_{\mathrm{bg}} + \frac{I_0}{\pi R(t)^2}\, U(R(t) - r)\,. \tag{S5}$$

Taking the temporal derivative gives two additive components: (i) a spatially uniform change within the disc due to the $R(t)^{-2}$ scaling, and (ii) a ring term supported only on the moving boundary $r = R(t)$:

$$\begin{aligned} I'(r,t) &= \left(\frac{I_0}{\pi R(t)^2}\right)' U(R(t) - r) + \frac{I_0}{\pi R(t)^2}\, R'(t)\, \delta(R(t) - r) \\ &= G(r,t) + H(r,t). \end{aligned} \tag{S6}$$

Here $\delta(\cdot)$ is the radial Dirac delta.

Evaluating the prefactors explicitly,

$$\begin{aligned} G(r,t) &= -\frac{2 I_0 R'(t)}{\pi R(t)^3}\, U(R(t) - r) \qquad \text{(uniform term inside the disc)}, \\ H(r,t) &= \frac{I_0}{\pi R(t)^2}\, R'(t)\; \delta(R(t) - r) \qquad \text{(Dirac ring at } r = R(t)\text{)}. \end{aligned} \tag{S7}$$

For compact notation (as in the notes), define scalar functions $g(t)$ and $h(t)$ such that

$$G(r,t) = g(t)\, U(R(t) - r)\,, \qquad H(r,t) = h(t)\, \delta(R(t) - r)\,. \tag{S8}$$

where

$$g(t) \equiv -\frac{2 I_0 R'(t)}{\pi R(t)^3}, \qquad h(t) \equiv \frac{I_0}{\pi R(t)^2}\, R'(t) \tag{S9}$$

## 1.4 Fourier-domain expressions (radial symmetry)

Assuming circular symmetry, the 2D spatial Fourier transform reduces to a zeroth-order Hankel transform. Let $k$ denote the radial spatial frequency. The transform of the uniform-disc derivative term is

$$\begin{aligned} \hat{G}(k,t) &= 2\pi \int_0^{\infty} G'(r,t)\, J_0(kr)\, r\, dr = 2\pi g(t) \int_0^{R(t)} J_0(kr)\, r\, dr \\ &= 2\pi g(t)\, \frac{R(t)\, J_1(kR(t))}{k}, \end{aligned} \tag{S10}$$

with the $k \to 0$ limit $G(0,t) = g(t)\pi R(t)^2$.

For the Dirac-ring term,

$$\begin{aligned}\hat{H}(k,t) &= 2\pi \int_0^\infty h(t)\,\delta(R(t)-r)\;J_0(kr)\,r\,dr \\ &= 2\pi h(t)\,R(t)\,J_0(kR(t))\,.\end{aligned} \tag{S11}$$

### 1.5 Dynamic deconvolution kernel

The dynamic deconvolution kernel (the Fourier transform of the PSF temporal derivative) is defined as the sum

$$D(k,t) \equiv \mathcal{F}\{I'(r,t)\} = \hat{G}(k,t) + \hat{H}(k,t), \tag{S12}$$

hence

$$D(k,t) = 2\pi R(t)\,g(t)\,\frac{J_1(kR(t))}{k} + 2\pi R(t)\,h(t)\,J_0(kR(t))\,. \tag{S13}$$

This $D(k,t)$ is the evolving PSF-derivative filter for a point source under the modulated ideal-lens model, and is the quantity used to construct the time-dependent deconvolution operator.

## 2 DATA AVAILABILITY

The data and figure reproduction for this work can be found on EventsLinearSystems_demo repository on GitHub: `https://github.com/nimrodkruger/EventsLinearSystems_demo`. Results in video form are uploaded to YouTube, and descriptions and links are provided as supplementary material.

## 3 ATTACHED VIDEO DESCRIPTION

Video files representing the Wiener deconvolution outcome for sampled event batches were uploaded to YouTube with the following links:

1. Single simulated point source: `https://youtube.com/shorts/04XVV5yrFcQ`
2. Two point source simulation with a distance of 5 pixels: `https://youtube.com/shorts/j-6O6EfGc2c`
3. Three subsections of an event-stream file of M47 star field recorded with a Prophesee Gen4 EVS camera, 0.5Hz modulation defocus frequency: `https://youtu.be/AXuxsw73Tbw`
